# Involvement of lipid rafts in adhesion-induced activation of Met and EGFR

**DOI:** 10.1186/1423-0127-18-78

**Published:** 2011-10-27

**Authors:** Ying-Che Lu, Hong-Chen Chen

**Affiliations:** 1 Graduate Institute of Biomedical Sciences, National Chung Hsing University, Taichung, Taiwan; 2 Department of Life Sciences, National Chung Hsing University, Taichung, Taiwan; 3 Argicultural Biotechnology Center, National Chung Hsing University, Taichung, Taiwan; 4 Department of Nutrition, China Medical University, Taichung, Taiwan

## Abstract

**Background:**

Cell adhesion has been shown to induce activation of certain growth factor receptors in a ligand-independent manner. However, the mechanism for such activation remains obscure.

**Methods:**

Human epidermal carcinoma A431 cells were used as a model to examine the mechanism for adhesion-induced activation of hepatocyte growth factor receptor Met and epidermal growth factor receptor (EGFR). The cells were suspended and replated on culture dishes under various conditions. The phosphorylation of Met at Y1234/1235 and EGFR at Y1173 were used as indicators for their activation. The distribution of the receptors and lipid rafts on the plasma membrane were visualized by confocal fluorescent microscopy and total internal reflection microscopy.

**Results:**

We demonstrate that Met and EGFR are constitutively activated in A431 cells, which confers proliferative and invasive potentials to the cells. The ligand-independent activation of Met and EGFR in A431 cells relies on cell adhesion to a substratum, but is independent of cell spreading, extracellular matrix proteins, and substratum stiffness. This adhesion-induced activation of Met and EGFR cannot be attributed to Src activation, production of reactive oxygen species, and the integrity of the cytoskeleton. In addition, we demonstrate that Met and EGFR are independently activated upon cell adhesion. However, partial depletion of Met and EGFR prevents their activation upon cell adhesion, suggesting that overexpression of the receptors is a prerequisite for their self-activation upon cell adhesion. Although Met and EGFR are largely distributed in 0.04% Triton-insoluble fractions (*i.e*. raft fraction), their activated forms are detected mainly in 0.04% Triton-soluble fractions (*i.e*. non-raft fraction). Upon cell adhesion, lipid rafts are accumulated at the cell surface close to the cell-substratum interface, while Met and EGFR are mostly excluded from the membrane enriched by lipid rafts.

**Conclusions:**

Our results suggest for the first time that cell adhesion to a substratum may induce a polarized distribution of lipid rafts to the cell-substratum interface, which may allow Met and EGFR to be released from lipid rafts, thus leading to their activation in a ligand-independent manner.

## Background

Aberrant activation of receptor tyrosine kinases (RTKs) is one of the major causes for malignant transformation [1]. Overexpression, mutation, or deletion of RTKs can facilitate their activation through a ligand-independent manner [2]. In particular, constitutive activation of epidermal growth factor receptor (EGFR) and/or hepatocyte growth factor receptor Met is often found in human malignancies, correlated with poor prognosis [3-5]. Cell-matrix adhesion has been shown to induce ligand-independent phosphorylation of Met and EGFR [6]. EGFR forms complexes with integrins upon cell adhesion, leading to phosphorylation of EGFR at specific tyrosine residues that are distinct from those caused by its ligands. In contrast, the phosphorylation of EGFR is abolished upon loss of cell adhesion [7,8]. Likewise, it was reported that the ligand-independent activation of Met relies on cell adhesion to fibronectin via α_5_β_1 _integrins [9]. However, the mechanism how cell adhesion activates both receptors remains poorly understood.

Lipid rafts are highly dynamic, nano-scaled, heterogeneous microdomains abundant in cholesterol and sphingolipid, which function to compartmentalize the plasma membrane [10]. Cell-matrix adhesion is involved in lipid rafts-mediated signal transduction pathways [11]. For example, integrin α6β4, a laminin receptor, is incorporated in lipid rafts through palmitoylation at cysteine in the membrane-proximal segment of β4 tail, which subsequently activates a palmitoylated Src family kinase in the rafts, important for mitogenic signalling [12]. Additionally, it has been demonstrated that integrin-mediated adhesion regulates the trafficking of lipid rafts components. Recently, RalA, a small GTPase, was identified as a key determinant for integrin-dependent membrane rafts trafficking and regulation of growth signalling [13].

In this study, we set out to examine the mechanism for adhesion-induced activation of Met and EGFR using human epidermal carcinoma A431 cells, in which EGFR and Met are overexpressed and constitutively activated. Possible involvement of matrix proteins, matrix stiffness, integrin β1, Src, reactive oxygen species (ROS), and the cytoskeleton were examined. However, none of these was found to be critical for adhesion-induced activation of Met and EGFR in A431 cells. Instead, we found for the first time that lipid rafts become accumulated at the cell-substratum interface, which may account, at least in part, for adhesion-induced activation of Met and EGFR.

## Methods

### Materials

Polyclonal anti-Met (C12), anti-EGFR (1005), and anti-ERK were purchased from Santa Cruz Biotechnology (Santa Cruz, CA). Monoclonal anti-EGFR pY1173 (#9H2), monoclonal anti-Met (DL-21), and polyclonal anti-integrin β1 (AB1952) were purchased from Millipore (Billerica, MA). Monoclonal anti-Met pY1234/1235 (D26), polyclonal anti-Met pY1349, and polyclonal anti-ERK pT202/Y204 were purchased from Cell Signaling Technology (Beverly, MA). Monoclonal anti-flotillin1 and Matrigel were purchased from BD Biosciences. Monoclonal anti-EGFR (ab30) was purchased from Abcam. Monoclonal anti-α-tubulin (DM1A), collagen I, poly-L-lysine (PLL), ethylene glycol-bis(2-aminoethylether)-N, N, N', N'-tetraacetic acid (EGTA), N-acetyl-L-cysteine (NAC), cytochalasin D, nocodazole, and polybrene were purchased from Sigma-Aldrich (St Louis, MO). The mouse ascites containing the monoclonal anti-Src (peptide 2-17) produced by hybridoma (CRL-2651) was prepared in our laboratory. Fibronectin, puromycin, and PHA665752 were purchased from Calbiochem (La Jolla, CA). Rhodamine-conjugated phalloidin and Alexa Fluor 488-conjugated cholera toxin subunit B (CTB-Alexa 488) were purchased from Invitrogen (Carlsbad, CA). Fetal bovine serum was purchased from Thermo Scientific HyClone (Logan, UT).

### Cell culture

A431 cells were maintained in Dulbecco's modified Eagle's medium (DMEM) supplemented with 10% fetal bovine serum and cultured at 37°C in a humidified atmosphere of 5% CO2 and 95% air. To examine adhesion-induced activation of Met and EGFR, A431 cells were seeded at 1.5 × 10^6 ^per 10-cm dish for 24 h, referred as attached cells. The attached cells were trypsinized, suspended in serum-free medium for 30 min, and then replated onto dishes coated with PLL or matrix proteins for 60 min before lysis. To examine the effect of cell-cell adhesion on ligand-independent activation of Met and EGFR, A431 cells were trypsinized and suspended at 2 × 10^5 ^cells/ml in serum-free DMEM with or without 2.5 mM EGTA. After constant rotation at 37°C for 24 h, the cells were lysed in 1% Nonidet P-40 lysis buffer and analysed by immunoblotting.

### Immunoblotting

Cells were lysed in 1% Nonidet P-40 lysis buffer (1% Nonidet P-40, 20 mM Tris-HCl, pH 8.0, 137 mM NaCl, 10% glycerol and 1 mM Na_3_VO_4_) containing protease inhibitors (1 mM phenylmethylsulfonyl fluoride, 0.2 trypsin inhibitory units/ml aprotinin, and 20 μg/ml leupeptin). The lysates were centrifuged for 10 minutes at 4°C to remove debris, and the protein concentrations were determined using the Bio-Rad protein assay (Hercules, CA). The total cell lysates were boiled for 3 minutes in SDS sample buffer, subjected to SDS-polyacrylamide gel electrophoresis, and transferred to nitrocellulose (Schleicher and Schuell). Immunoblotting was performed with appropriate antibodies using the Millipore enhanced chemiluminescence system for detection. Chemiluminescent signals were detected and quantified by Fuji LAS-3000 luminescence image system.

### Lentiviral production and infection

The lentiviral system for short-hairpin RNA (shRNA) was provided by the National RNAi Core Facility, Academia Sinica, Taiwan. To produce lentiviruses, HEK293T cells were co-transfected with 2.25 μg pCMV-ΔR8.91, 0.25 μg pMD.G, and 2.5 μg hairpin-pLKO.1 by TransIT-LT1 (Mirus Bio). After 3 days, the medium containing lentiviral particles was collected and stored at -80°C. The cells were infected with recombinant lentiviruses encoding shRNAs in the medium supplemented with 8 μg/ml polybrene (Sigma-Aldrich) for 24 h. Subsequently, the cells were selected in the growth medium containing 0.4 μg/ml puromycin and the puromycin-resistant cells were collected for analysis.

### Matrigel invasion assay

The 24-well transwell chambers (Costar) separated by a membrane with 8-μm pores were coated with 100 μl Matrigel (1.6 mg/ml). The lower chamber was loaded with 750 μl DMEM with 10% serum. The cells were added to the upper chamber in 250 μl serum-free medium. After 24 h, the cells that had migrated through Matrigel were stained by Giemsa stain and counted.

### Preparation of 2.8 kPa polyacrylamide gel

30% (w/v) acrylamide and 1% (w/v) bis-acrylamide were prepared as described previously [14,15]. To prepare a polyacrylamide gel with elastic moduli of 2.8 kPa, acrylamide and bis-acrylamide at the final concentrations of 7.5% and 0.1%, respectively, were allowed to be polymerized by addition of TEMED and 10% ammonium persulfate. The Mini-PROTEAN III (Bio-Rad) was used to cast the polyacrylamide gel. When the polymerization is completed, the gel were transferred to cell culture dishes and immersed in phosphate-buffered saline (PBS) for overnight.

### Cellular fractionation

The confluent A431 cells in 10-cm dishes were washed three times with ice-cold PBS and scraped into buffer A (150 mM NaCl, 1 mM EDTA, 50 mM Tris-HCl pH 7.4, 1 mM PMSF, 5 μg/ml aprotinin) containing 0.04% Triton X-100 with gentle mixing at 4°C for 10 min. The lysates were centrifugated at 14,000 × g for 20 min at 4°C, and the supernatant was transferred to a new eppendorf tube. This fraction is referred as soluble fraction. The insoluble pellets were resuspended in buffer A containing 1% Triton X-100 for 30 min on ice. Debris was pelleted after centrifugation at 14,000 × g for 20 min at 4°C, and the supernatant was collected as insoluble fraction.

### Confocal fluorescence microscopy and total internal reflection fluorescence microscopy

To stain cell surface Met or EGFR, cells were fixed by 4% paraformaldehyde in PBS for 30 min at room temperature, stained with anti-Met (DL-21) or monoclonal anti-EGFR (ab30) at 4°C overnight, and followed by Alexa 488-conjugated or Alexa 546-conjugated secondary antibodies for 1 h at room temperature. To stain lipid rafts, cells were rinsed with chilled growth medium then incubated with 1 μg/ml CTB-Alexa 488 at 4°C for 15 min before fixation in 4% paraformaldehyde. Rhodamine-conjugated phalloidin were used to stain actin filaments. Coverslips were mounted in anti-Fade DAPI-Fluoromount-G™ (SouthernBiotech; Birmingham, AL) and viewed using a Zeiss LSM510 laser scanning confocal microscope image system with a Zeiss 100X Plan-Apochromat objective (NA 1.4 oil). For total internal reflection fluorescence microscopy, the coverslips were viewed using an inverted Zeiss microscope (Axio Observer D1) with α Plan-Fluar 100X/1.45 III objective.

### Statistics

Statistical analyses were performed with Student's *t *test. Differences were considered to be statistically significant at *P <*0.05.

## Results

### Ligand-independent activation of Met and EGFR confers proliferative and invasive advantages to A431 cells

**Figure 1:**
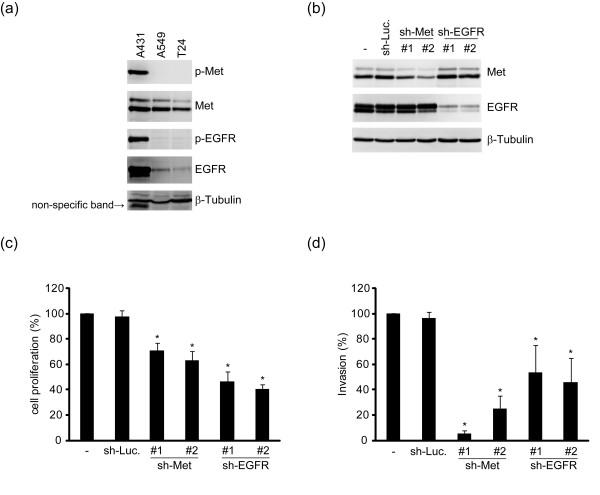
**Ligand-independent activation of Met and EGFR confers proliferative and invasive advantages to A431 cells**. **(a) **An equal amount of whole cell lysates from epidermal carcinoma A431 cells, lung adenocarcinoma A549 cells, and bladder carcinoma T24 cells was analyzed by immunoblotting with antibodies as indicated. The activation of Met and EGFR were detected by anti-Met pY1234/1235 (p-Met) and anti-EGFR pY1173 (p-EGFR). **(b) **An equal amount of whole cell lysates from the A431 cells stably expressing shRNAs specific to Met (sh-Met clone #1 and #2), EGFR (sh-EGFR clone #1 and #2) or luciferase (sh-Luc) was analyzed by immunoblotting with anti-Met and anti-EGFR. β-tubulin was used as an internal control. **(c) **The cells as described in the panel (b) were seeded on 6-cm dishes and the cell number was counted after 4 days. Data are quantified and expressed as percentage relative to the level of the control A431 cells, which is defined as 100%. Values (means ± s.d.) are from three independent experiments. *p < 0.05 (compared with the sh-Luc). **(d) **The cells as described in the panel (b) were subjected to a Matrigel invasion assay. Data are quantified and expressed as percentage relative to the level of the control A431 cells, which is defined as 100%. Values (means ± s.d.) are from three independent experiments. *p < 0.05 (compared with the sh-Luc)

Ligand-independent activation of RTKs as a result of gene mutation or overexpression is a hallmark of malignant transformation [1]. Overexpression and/or mutation in Met and EGFR have been implicated in the etiology of a variety of human tumors [5,16]. In this study, we found that both Met and EGFR are constitutively activated in human epidermal A431 cells (Figure 1a). Depletion of either Met or EGFR in the cells had adverse effects on their proliferation and invasiveness (Figures 1b-d). However, the cell proliferation appeared to rely more on EGFR than on Met (Figure 1c). On the other hand, the invasiveness of the cells depended more on Met than on EGFR (Figure 1d). These results suggest that ligand-independent activation of Met and EGFR may preferentially contribute to different aspect of malignant transformation.

### Ligand-independent activation of Met and EGFR in A431 cells relies on cell attachment to a substratum, but independent of cell spreading, ECM proteins, and substratum stiffness

**Figure 2:**
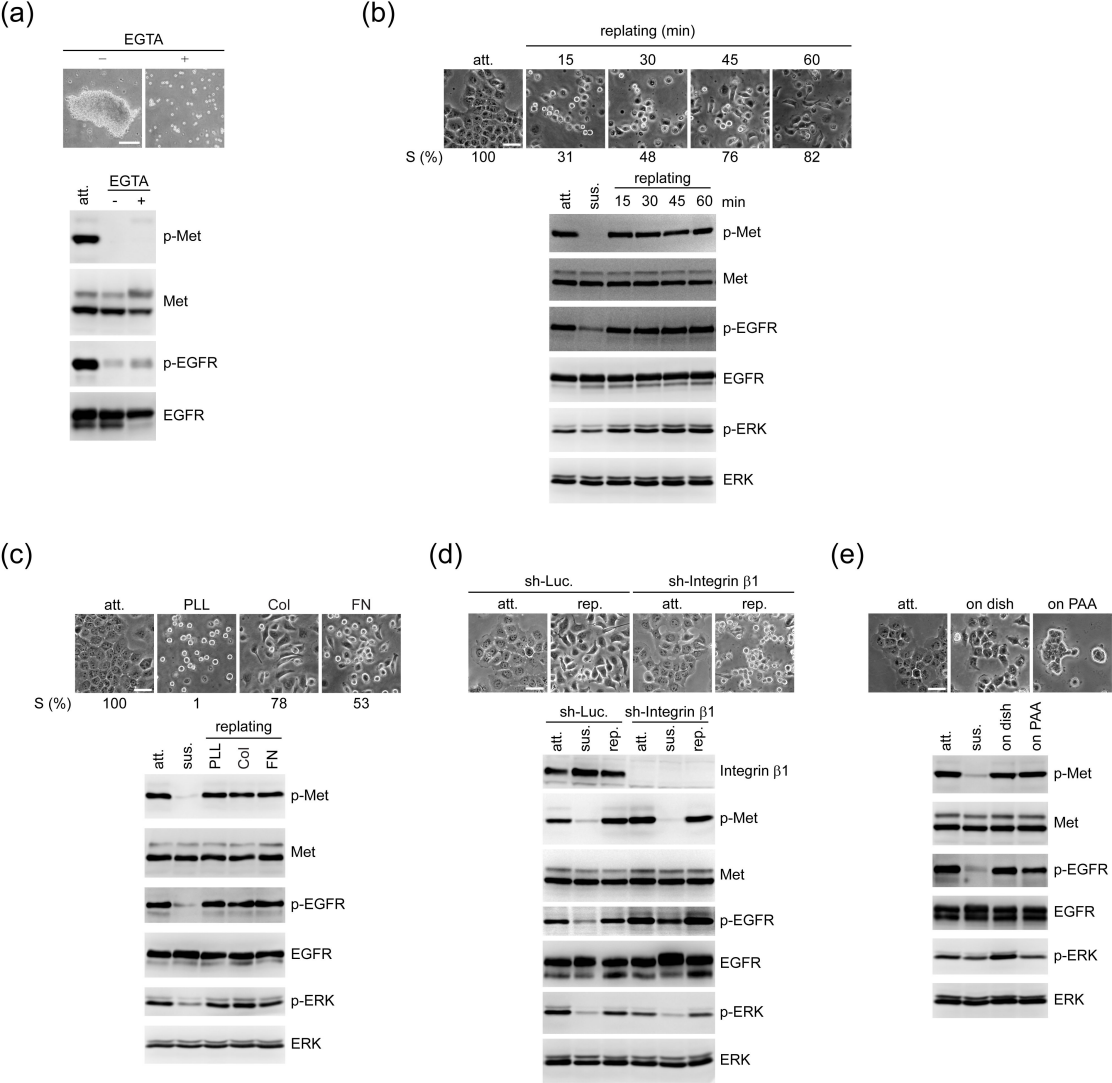
**Cell adhesion induces ligand-independent activation of Met and EGFR**. **(a) **A431 cells were suspended in serum-free medium with (+) or without (-) 2.5 mM EGTA with constant rotation for 24 hours. Representative images from suspended cells are shown. Scale bar, 150 μm. An equal amount of whole cell lysates was analysed by immunoblotting. **(b) **A431 cells were seeded on culture dishes for 24 h, referred as attached cells (att). The attached cells were trypsinized, suspended in serum-free medium for 30 min, and lyzed (sus). For replating experiments, the suspended cells were replated on dishes coated with 10 μg/ml collagen for various times. The percentage of cell spreading (S) in total counted cells (n ≥ 200) was measured. Representative images are shown. Scale bar, 50 μm. An equal amount of whole cell lysates was analysed by mmunoblotting with antibodies as indicated. **(c) **A431 cells were suspended and then replated on dishes coated with 100 μg/ml poly-L-lysine (PLL), 10 μg/ml collagen (Col), or fibronectin (FN) for 60 min. The percentage of cell spreading (S) in total counted cells (n ≥ 200) was measured. Representative images are shown. Scale bar, 50 μm. **(d) **The attached A431 cells stably expressing shRNAs specific to integrin β1 (sh-Integrin β1) or luciferase (sh-Luc) were suspended and replated on dishes coated with collagen for 60 min. An equal amount of whole cell lysates from attached (att), suspended (sus), and replated (rep) cells was analysed by immunoblotting with antibodies as indicated. Scale bar, 50 μm. **(e) **A431 cells were suspended and then replated onto a culture dish (on dish) or a layer of polyacrylamide gel (on PAA) with elastic moduli of 2.8 kPa, which mimics the stiffness of soft tissues, in the medium with 1% serum for 3 h. Representative images are shown. Scale bar, 50 μm.

When the transforming potential of A431 cells was measured, we noted that A431 cells fail to grow in soft agar (data not shown). This phenomenon implicated that the ligand-independent activation of Met and EGFR in A431 cells might rely on cell adhesion. Indeed, both Met and EGFR in A431 cells were no longer retained in the active state when the cells were kept in suspension (Figure 2a). In the absence of EGTA, the cells were allowed to aggregate and form large cell masses in suspension. However, even under such a condition, Met and EGFR were not activated, indicating that ligand-independent activation of Met and EGFR in A431 cells relies on cell adhesion to a substratum rather than cell-cell adhesions.

We reasoned such adhesion-induced activation of Met and EGFR in A431 cells might be related to cell spreading, cell-matrix interaction, or stiffness of substrata. To examine the effect of cell spreading, A431 cells were seeded on culture dishes for 24 hours, referred as attached cells. To collect cell lysates from suspended cells, the attached cells were trypsinized, suspended in serum-free medium, and lyzed. For replating experiments, the suspended cells were replated on dishes coated with collagen or poly-L-lysine (PLL) for various intervals. Fifteen min after plating on collagen, the activation of Met and EGFR reached the maximum (Figure 2b). Longer incubation allowed more cells to spread, but it did not induce more activation in Met and EGFR. Additionally, the extent of Met and EGFR activation induced by cell adhesion to PLL was similar to that induced by cell adhesion to matrix proteins including collagen and fibronectin (Figure 2c). These results together indicate that adhesion-induced activation of Met and EGFR in A431 cells is independent of cell spreading or cell-matrix interaction. In consistent with this notion, we found that although shRNA-mediated depletion of integrin β1 in A431 cells severely impaired cell-matrix adhesion, it does not affect activation of Met and EGFR upon cell attachment (Figure 2d). Next, the effect of substratum stiffness was evaluated. A431 cells were replated to a polyacrylamide gel which mimics the stiffness of soft tissues, as described previously [14,15]. Our results showed that attachment of A431 cells to the polyacrylamide gel induced activation of Met and EGFR to an extent similar to that induced by cell attachment to culture dishes (Figure 2e). Our results thus suggest that substratum stiffness is not a determinant for Met and EGFR activation in A431 cells.

### Adhesion-induced activation of Met and EGFR in A431 cells is independent of Src, ROS, or cytoskeleton

**Figure 3:**
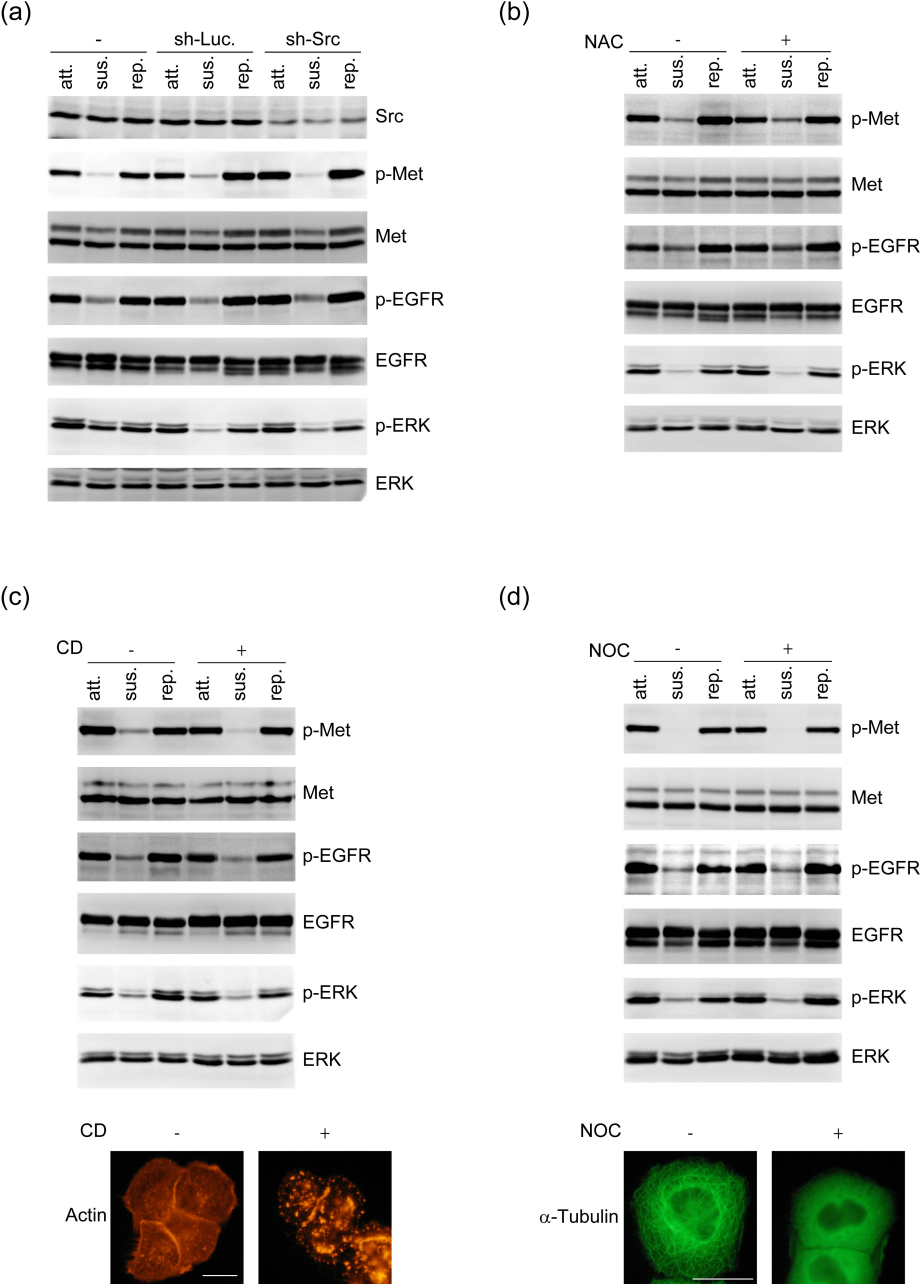
**Adhesion-induced activation of Met and EGFR does not rely on Src, ROS, or cytoskeleton**. **(a) **The control A431 cells and those stably expressing shRNAs specific to Src (sh-Src) or luciferase (sh-Luc) were kept in suspension for 30 min and replated on dishes coated with collagen for 60 min. An equal amount of whole cell lysates from attached (att), suspended (sus), and replated (rep) cells was analysed by immunoblotting with antibodies as indicated. **(b) **An equal amount of whole cell lysates from attached (att), suspended (sus), and replated (rep) A431 cells in the presence (+) or absence (-) of 30 mM NAC for 30 min was analysed by immunoblotting with antibodies as indicated. **(c) **An equal amount of whole cell lysates from attached (att), suspended (sus), or replated (rep) A431 cells in the presence (+) or absence (-) of 2 μM cytochalasins D (CD) for 60 min was analysed by immunoblotting with antibodies as indicated. The cells were stained for F-actin with phalloidin. Representative images for F-actin staining are shown. Scale bar, 20 μm. **(d) **An equal amount of whole cell lysates from attached (att), suspended (sus), or replated (rep) A431 cells in the presence (+) or absence (-) of 30 μg/ml nocodazole (NOC) for 60 min was analysed by immunoblotting with antibodies as indicated. The cells were stained for microtubules with anti-α-tubulin. Representative images are shown. Scale bar, 20 μm.

Adhesion-induced activation of RTKs has been attributed to Src activation [8,17]. However, this is not the case in A431 cells, because neither Src knockdown (Figure 3a) nor Src inhibitors (data not shown) affected Met and EGFR activation upon cell adhesion. Next, we suspected that reactive oxygen species (ROS) or the cytoskeleton might be involved. Elimination of intracellular ROS by NAC (Figure 3b) or disruption of actin filaments by cytochalasin D (Figure 3c) and microtubules by nocodazole (Figure 3d) did not prevent Met and EGFR activation upon cell adhesion. Therefore, the possibilities for Src, ROS, and the integrity of cytoskeleton to play a key role in adhesion-induced activation of Met and EGFR were excluded.

### Met and EGFR are independently activated upon cell adhesion

**Figure 4:**
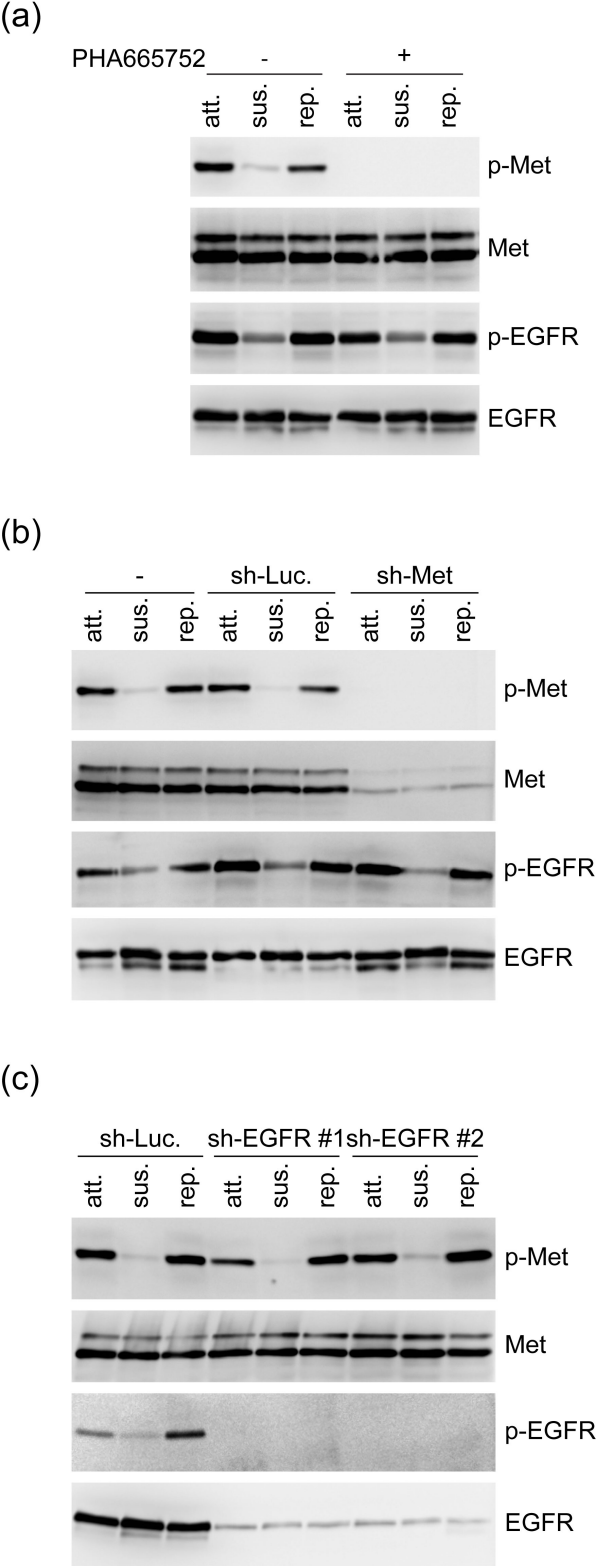
**Met and EGFR in A431 cells are independently activated upon cell adhesion**. **(a) **An equal amount of whole cell lysates from attached (att), suspended (sus), and replated (rep) A431 cells in the presence (+) or absence (-) of 0.5 μM PHA665752 (a specific inhibitor for Met) for 60 min was analysed by immunoblotting with antibodies as indicated. **(b) **The control A431 cells and those stably expressing shRNAs specific to Met (sh-Met) or luciferase (sh-Luc) were kept in suspension for 30 min and replated on dishes coated with collagen for 60 min. An equal amount of whole cell lysates from attached (att), suspended (sus), and replated (rep) cells was analysed by immunoblotting with antibodies as indicated. **(c) **The A431 cells stably expressing shRNAs specific to EGFR (sh-EGFR clone #1 and #2) or luciferase (sh-Luc) were suspended in serum-free medium for 30 min and then replated on dishes coated with collagen for 60 min. An equal amount of whole cell lysates from attached (att), suspended (sus), and replated (rep) cells was analysed by immunoblotting with antibodies as indicated.

As Met and EGFR have been reported to physically interact with and activate each other [18-20], we examined if adhesion-induced activation of Met and EGFR in A431 cells relies on their mutual activation. We found that suppression of Met by the specific inhibitor PHA665752 or shRNA did not affect EGFR activation upon cell adhesion (Figures 4a and 4b). In addition, depletion of EGFR did not affect Met activation either (Figure 4c). Thus, these results indicate that Met and EGFR in A431 cells are not reciprocally activated upon cell adhesion.

### Overexpression of Met and EGFR is necessary for their activation upon cell adhesion

**Figure 5:**
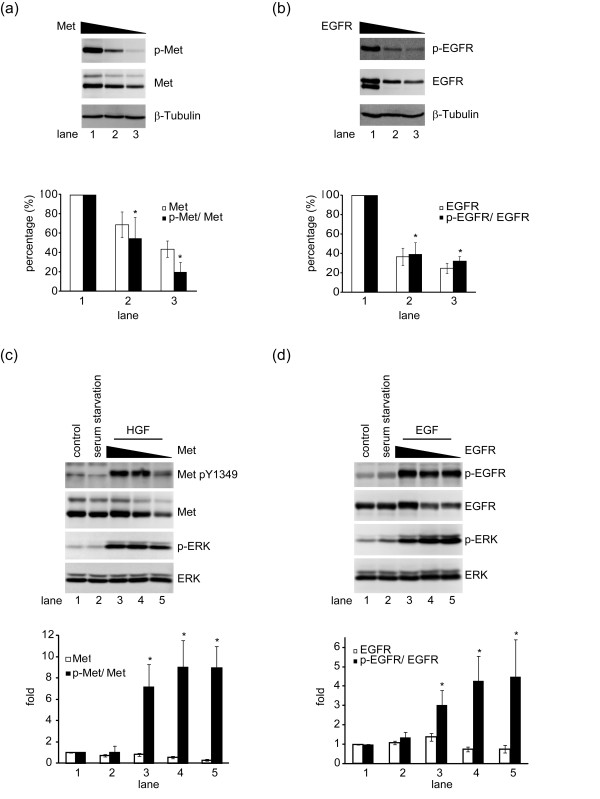
**Overexpression of Met and EGFR is necessary for their activation upon cell adhesion**. **(a) **The expression of Met in A431 cells was reduced to different levels by shRNA. The adhesion-induced activation of Met was analysed by normalization of phospho-Met (p-Met) to its expression level (Met). β-tubulin was used as an internal control. Data (Met and p-Met/Met) are quantified and expressed as percentage relative to the level of the control A431 cells, which is defined as 100%. Values (means ± s.d.) are from three independent experiments. **(b) **The expression of EGFR in A431 cells was reduced to different levels by shRNA. The adhesion-induced activation of EGFR was analysed by normalization of phospho-EGFR (p-EGFR) to its expression level (EGFR). β-tubulin was used as an internal control. Data (EGFR and p-EGFR/EGFR) are quantified and expressed as percentage relative to the level of the control A431 cells, which is defined as 100%. Values (means ± s.d.) are from three independent experiments. **(c) **The cells as described in (a) were serum-starved for 24 h and treated with HGF (40 ng/ml) for 10 min. The ligand-induced activation of Met was analysed as described in (a). Data (Met and p-Met/Met) are quantified and expressed as percentage relative to the level of the control A431 cells, which is defined as 100%. Values (means ± s.d.) are from three independent experiments. **(d) **The cells as described in (b) were serum-starved for 24 h and treated with EGF (100 ng/ml) for 10 min. The ligand-induced activation of EGFR was analysed as described in (b). Data (EGFR and p-EGFR/EGFR) are quantified and expressed as percentage relative to the level of the control A431 cells, which is defined as 100%. Values (means ± s.d.) are from three independent experiments.

Ligand-independent activation of RTKs has been attributed to their overexpression [21,22]. To examine whether overexpression of Met and EGFR in A431 cells is necessary for their activation upon cell adhesion, the expression of Met or EGFR in A431 cells was suppressed by specific shRNAs. To compare the activation of the receptors, the phosphorylation of the receptors was normalized to their expression level. When the expression of Met or EGFR was reduced, the extent of Met and EGFR activation was decreased (Figures 5a and 5b). For further examination of the responsiveness to ligand stimulation, the cells were serum starved for 24 hours and treated with HGF or EGF for 10 min. However, the reduced level of Met and EGFR retained their responsiveness to ligand stimulation (Figures 5c and 5d). Therefore, ligand-independent activation of Met and EGFR in A431 cells relies on their overexpression in order for them to be self-activated.

### Accumulation of lipid rafts to the cell-substratum interface may allow activation of Met and EGFR

**Figure 6:**
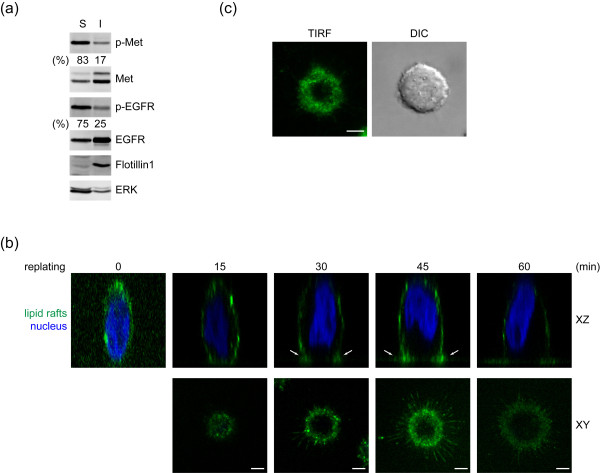
**Accumulation of lipid rafts at the cell-substratum interface upon cell adhesion**. **(a) **A431 cells were fractionated as described in Methods. An equal portion of cell lysates from soluble and insoluble fractions was analysed by immunoblotting with antibodies as indicated. Flotillin1 is used as a marker for lipid rafts. ERK is used as a marker for non-rafts. The phosphorylation of Met and EGFR was measured. Data shown are representative from two independent experiments. **(b) **A431 cells were kept in suspension for 30 min and replated on glass coverslips coated with 100 μg/ml poly-L-lysine for various time as indicated. The cells were stained for lipid rafts with Alexa 488-conjugated cholera toxin B subunit and visualized by confocal microscopy. Representative *XZ *and *XY *images at indicated time are shown. The XY sections were taken at the height of 3.5 μm above the bottom. Scale bar, 5 μm. **(c) **A431 cells were replated on glass coverslips coated with 100 μg/ml poly-L-lysine for 30 min, stained for lipid rafts with Alexa 488-conjugated cholera toxin B subunit, and visualized by total internal reflection fluorescence (TIRF) microscopy. The morphology of the same cell was taken by a differential interference contrast (DIC) objective. Scale Bar, 5 μm.

**Figure 7:**
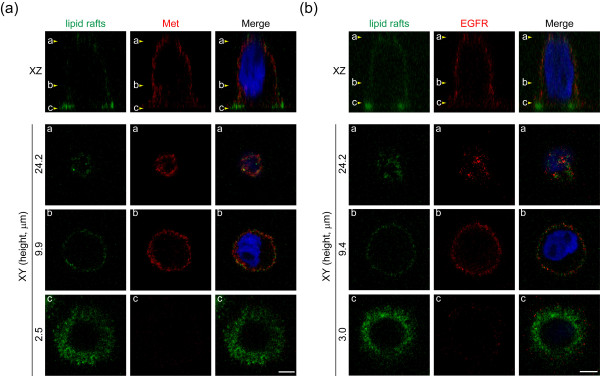
**Met and EGFR are excluded from the plasma membrane enriched by lipid rafts upon cell adhesion**. A431 cells were replated on glass coverslips coated with 100 μg/ml poly-L-lysine for 30 min and stained for lipid rafts with Alexa 488-conjugated cholera toxin B subunit. The cells were stained for Met (**a**) or EGFR (**b**) with a monoclonal antibody which specifically recognizes the extracellular domain of the receptors and visualized by confocal microscopy. Arrowheads on the *XZ-*section images indicate the height at which *XY *-section images were taken. Scale Bar, 5 μm.

Lipid rafts have been shown to involve in regulation of RTK activation [11]. In A431 cells, we found that active forms of Met and EGFR were mainly detected in the 0.04% Triton-soluble fraction (Figure 6a), suggesting that Met and EGFR are likely to be activated in non-lipid rafts. To visualize the distribution of lipid rafts at the plasma membrane, A431 cells were stained with Alexa488-conjugated cholera toxin B subunit, which binds to ganglioside GM1, a lipid-raft marker [23]. Lipid rafts were distributed randomly at the plasma membrane in suspended cells (Figure 6b). Surprisingly, upon cell adhesion, lipid rafts became accumulated at the cell surface close to the cell-substratum interface (Figure 6b), while Met and EGFR were mostly excluded from the areas enriched by lipid rafts (Figures 7a and 7b). The fluorescent signals for lipid rafts were confirmed to be close to the substratum by a total internal refection fluorescence microscope, which detect signals ~200 nm above the coverslip (Figure 6c). These data suggest that cell adhesion may induce a polarized distribution of lipid rafts to the cell-substratum interface, which may allow Met and EGFR to stay out of lipid rafts, leading to their activation in a ligand-independent manner.

## Discussion

Ligand-independent activation of RTKs by cell adhesion can be important for tumor progression. Upon adhesion to extracellular matrixes in the tumor microenvironment, tumor cells may acquire growth potential because of RTK activation. Besides, once tumor cells invade into blood vessels, they need to attach to endothelial cells for extravasation. This tumor-endothelium contact might be sufficient to induce a ligand-independent activation of RTKs, rendering advantages for tumor cell survival and metastasis. In this study, we show that ligand-independent activation of EGFR and Met is important for proliferation and invasiveness of A431 cells (Figure 1) and demonstrate such activation relies on cell adhesion to a substratum (Figure 2a). In our efforts to understand the underlying mechanism for such activation, we have excluded the significance of cell spreading, matrix proteins, matrix stiffness, Src, ROS, and cytoskeletal integrity in this process (Figures 2 and 3). Although integrins have been reported to be important for RTK activation [24], two lines of evidence from our study indicate that the integrin-matrix interaction is not responsible for adhesion-induced activation of Met and EGFR, at least, in A431 cells. First, cell adhesion to poly-L-lysine is sufficient to activate Met and EGFR (Figure 2c). Second, depletion of integrin β1 by shRNA does not impair the attachment-induced activation of Met and EGFR (Figure 2d). However, although our data support that the initial activation of Met and EGFR upon cell adhesion does not require the integrin-matrix interaction, it remains possible that the integrin-matrix interaction may contribute to sustained activation of Met and EGFR.

We found in this study that lipid rafts are randomly distributed on the plasma membrane of suspended cells, which are recruited to the ventral surface of the cells upon cell attachment (Figures 6b and 6c). This is likely to be a general phenomenon, because it is observed not only in A431 cells but also in other types of cells such as SW620 and A549 cells (data not shown). The lipid rafts enriched at the cell-substratum interface display a donut-like shape with a diameter of 10-25 μm. This recruitment can be observed as early as 15 min after cell adhesion and reach to a plateau at 30-45 min (Figure 6b). Along with cell spreading, the lipid rafts become less accumulated at the ventral surface and eventually distributed more evenly on the whole cell surface (Figure 6b). Intriguingly, once cell-cell adhesions are formed, lipid rafts become accumulated at cell-cell junctions, in particular, tight junctions (data not shown). Therefore, it is apparent that lipid rafts are dynamic, whose distribution at the plasma membrane can be dramatically changed according to the status of cell adhesions, implicating their roles in various processes. However, the driving force for lipid rafts to accumulate at a particular subdomain of the plasma membrane remains unknown.

As Met and EGFR are mostly activated in the non-rafts fraction (Figure 6a), it is reasonable to speculate that the microenvironment within lipid rafts does not favor the activation of both receptors. Although the mechanism by which lipid rafts keep Met and EGFR inactive is not clear, it is possible that the low membrane fluidity of lipid rafts may prevent their self-association in order to form dimers or oligomers. Alternatively, certain negative regulators of Met and EGFR may be present in lipid rafts, which bind and inhibit the activation of the receptors. In this study, we found that while lipid rafts are accumulated at the ventral surface close to the cell-substratum interface, Met and EGFR are mostly excluded from the membrane enriched by lipid rafts (Figure 7). Therefore, the accumulation of lipid rafts at the ventral surface may be important for the activation of Met and EGFR. We assume that the accumulation of lipid rafts to the cell-substratum interface may allow Met and EGFR to be released from lipid rafts, leading to their activation in the non-raft membrane. However, how lipid rafts become accumulated at the cell-substratum interface upon cell adhesion and how Met and EGFR can be excluded from the raft-enriched membrane upon cell adhesion are currently unknown. It has been demonstrated that cholesterol accumulation, which breaks down the homeostasis of the plasma membrane, leads to coalescence of lipid rafts and induces a more malignant phenotype in prostate cancers [25,26]. Therefore, it is evident that the consitution and distribution of lipid rafts are closely linked to RTK activaiton and cellular trasnformation.

## Conclusions

Ligand-independent activation of Met and EGFR in A431 cells relies on cell attachment to a substratum, but is independent of cell spreading, extracellular matrix proteins, and substratum stiffness. This cell attachment-induced activation of Met and EGFR cannot be attributed to Src activation, ROS production, and the integrity of the cytoskeleton. Instead, overexpression of the receptors is a prerequisite for their self-activation upon cell attachment. Moreover, we demonstrate that Met and EGFR are independently activated upon cell attachment. Finally, sequestration of lipid rafts at the cell-substratum interface may allow Met and EGFR to be activated in the non-raft membrane upon cell adhesion.

## Competing interests

The authors declare that they have no competing interests.

## Authors' contributions

YCL carried out all experiments in the study and helped to draft the manuscript. HCC conceived of the study and participated in its design and coordination. All authors read and approved the final manuscript.
